# Orphan Legumes Growing in Dry Environments: Marama Bean as a Case Study

**DOI:** 10.3389/fpls.2018.01199

**Published:** 2018-08-15

**Authors:** Christopher Cullis, Percy Chimwamurombe, Nigel Barker, Karl Kunert, Juan Vorster

**Affiliations:** ^1^Department of Biology, Case Western Reserve University, Cleveland, OH, United States; ^2^Department of Natural and Applied Sciences, Namibia University of Science and Technology, Windhoek, Namibia; ^3^Department of Plant and Soil Sciences, University of Pretoria, Pretoria, South Africa; ^4^Department of Plant and Soil Sciences, Forestry and Agricultural Biotechnology Institute, University of Pretoria, Pretoria, South Africa

**Keywords:** orphan legumes, marama bean, drought response, soil microbiome, genome sequence

## Abstract

Plants have developed morphological, physiological, biochemical, cellular, and molecular mechanisms to survive in drought-stricken environments with little or no water caused by below-average precipitation. In this mini-review, we highlight the characteristics that allows marama bean [*Tylosema esculentum* (Burchell) Schreiber], an example of an orphan legume native to arid regions of southwestern Southern Africa, to flourish under an inhospitable climate and dry soil conditions where no other agricultural crop competes in this agro-ecological zone. Orphan legumes are often better suited to withstand such harsh growth environments due to development of survival strategies using a combination of different traits and responses. Recent findings on questions on marama bean speciation, hybridization, population dynamics, and the evolutionary history of the bean and mechanisms by which the bean is able to extract and conserve water and nutrients from its environment as well as aspects of morphological and physiological adaptation will be reviewed. The importance of the soil microbiome and the genetic diversity in this species, and their interplay, as a reservoir for improvement will also be considered. In particular, the application of the newly established marama bean genome sequence will facilitate both the identification of important genes involved in the interaction with the soil microbiome and the identification of the diversity within the wild germplasm for genes involved drought tolerance. Since predicted future changes in climatic conditions, with less water availability for plant growth, will severely affect agricultural productivity, an understanding of the mechanisms of unique adaptations in marama bean to such conditions may also provide insights as to how to improve the performance of the major crops.

## Dry Environments and Orphan Legumes

Dry environments have little or no water with below-average precipitation due to periodic droughts resulting in prolonged water shortage. A drought period can last from a few days to months or years. Such a drought period is often accompanied by intensive heat significantly worsening drought effects due to additional increased water evaporation. Drought-stricken environments are highly unsuitable for production of high-input, high-yielding food crops mostly selected for optimal yield under non-drought conditions (Evenson and Gollin, 2003).

Orphan, or underutilized, legumes, are staple food crops in many developing countries. Their neglect has been the subject of two reports from the National Research Council (1979, 2006). They have generally little economic importance and have not been greatly improved by breeders (Naylor et al., 2004; Foyer et al., 2016). Like landraces of major food crops, orphan legumes have genetically adapted over time to their natural environment largely unmodified by human breeding efforts (Trytsman et al., 2011). These legumes are often better suited to withstand harsh growth environments, having developed morphological, physiological, biochemical, cellular and molecular mechanisms to survive in dry drought-stricken environments (Osakabe et al., 2014; Tátrai et al., 2016). Although orphan legumes frequently respond like most plants to stress, they might have developed unique survival mechanisms using a combination of different traits and responses resulting in growth in dry drought-stricken environments. Detailed knowledge of such mechanisms therefore provides valuable information for breeders on useful traits and responses for survival in extreme environments (Cullis and Kunert, 2017). Such orphan legume crops include groundnut (*Arachis hypogaea*), grass pea (*Lathyrus sativus*), bambara groundnut (*Vigna subterranea*), cowpea (*Vigna unguiculata*), and marama bean (*Tylosema esculentum*), the last included by The Kirkhouse Trust focusing on improving locally important legume crops^1^.

Purpose of this mini-review is to provide a short overview of the existing knowledge and current advances in the research to understand the biology of the plant. This also includes the mechanisms the bean employs to survive in regions where few conventional crops can thrive and flourish. An improved understanding of these mechanisms might also not only help breeders to learn more about particular characteristics for survival in dry drought stricken environments but also the bean’s usefulness, both as a model and a future crop, to combat climate change and to attract interest in the development of the bean into mainstream agriculture.

## Marama Bean, a Pea Family Member

Marama bean, a wild perennial legume native to the Kalahari Desert in Southern Africa, grows mostly in sandy soils. Reports of the National Research Council, 1979 on “Tropical Legumes: Resources for the Future” and also in 2006 on “Lost Crops of Africa: Volume II: Vegetables” highlight the bean. In 1979, it was for example noted that “Of all the plants described in this book, the marama bean is, perhaps, the least developed,” while in 2006 it was noted “Strange that marama has not been introduced into cultivation since above ground, this plant produces seeds that rival peanut and soybean in composition and nutritive value, and below, it produces a high-protein tuber much bigger and more nutritious than any potato, yam, or even sugar beet. In addition, it thrives in poor-quality soil and under the harshest of climates. Little is known about the plant and almost nothing is understood about its cultivation. Among Africa’s many native foods, this remains one of the most neglected.”

**FIGURE 1:**
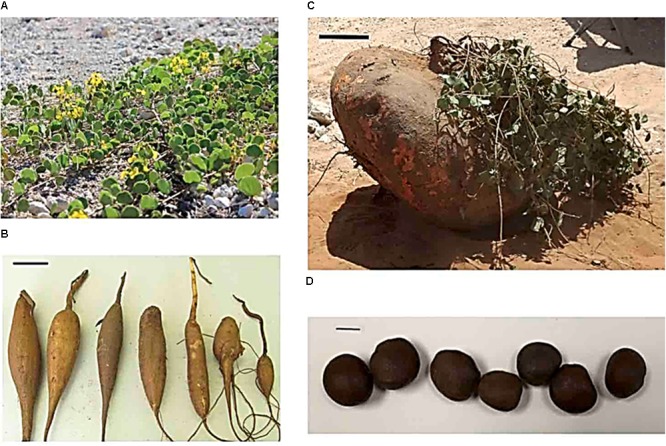
**(A)** Prostrate vines with flowers. **(B)** Young and edible marama bean tuberous roots. Scale bar in **A** is 10 cm. **(C)** Large tuber weighing approximately 240 kg, with abundant foliage. Scale bar in **C** is 25 cm. **(D)** Seeds. Scale bar in **D** is 1 cm.

The bean is an important local dietary component, due to its high seed protein and high carbohydrate content of the tuberous root (**Figure 1**). In its native habitat, the bean withstands summer temperatures reaching 50°C with surface water available usually only for 8 weeks/year (Powell, 1987; Bower et al., 1988; Nepolo et al., 2009). Marama bean is currently being developed into a local crop (Chimwamurombe, 2011) and test gardens have been already established and efforts are undertaken in Namibia to produce the bean in well-fenced prepared land for local communities.

Marama bean belongs to the subfamily Cercidoideae of the Fabaceae (pea family). The genus *Tylosema*, to which the bean belongs, has four additional species (Lewis et al., 2005; Azani et al., 2017). All members of the Cercidoideae subfamily lack root nodules. *Tylosema* species have been investigated by both palynological and molecular analyses particularly by application with chloroplast markers such as the matK gene (Hao et al., 2003; Banks et al., 2013). A further member of the subfamily Cercidoideae is *Bauhinia*, a genus of more than 500 species of flowering plants widely cultivated as ornamental trees in tropical Asia. *Tylosema* was not considered as a distinct genus and was originally classified within the *Bauhinia* genus (Hao et al., 2003). However, *Tylosema*, was later recognized as a separate clade within the *Bauhiniinae* (Wunderlin, 2010). Separation between *Tylosema* and *Bauhinia* has recently been confirmed by complete chloroplast genomic sequencing and by comparing specific sequences of a number of genes both from the chloroplast and nucleus (Kim and Cullis, 2017). *T. esculentum*, *Tylosema fassoglense*, and putative Angolan species (Castro et al., 2005) may be, in reality, a single genetically diverse species, within which it maybe be possible to identify land races or some similar entity. As with many African plant lineages, marama bean fits nicely with the little used “ochlospecies” concept first proposed by White (1998), and which seems to apply to many widespread African taxa (Cronk, 1998).

## Drought Adaptation Mechanisms

Marama bean has developed, as a drought avoider, several survival mechanisms for life in a dry drought-stricken environment. The morphological and physiological adaptation of the bean to its growth environment has been recently reviewed by Lawlor (2018). The bean reacts like many other plants growing in dry environments but combines different well-known avoidance mechanisms. The bean avoids water stress by leaf reduction to reduce water loss and reduces stem elongation and number of leaves, at the extreme to complete die back in cooler months (Mitchell et al., 2005; Travlos and Karamanos, 2008; Karamanos and Travlos, 2012). Under well-watered conditions, the bean is highly branched with runners extending along the ground and produces a great number of leaves and biomass (Mitchell et al., 2005; Karamanos and Travlos, 2012; **Figure 1A**). The bean has also a tap root allowing penetration deep below the surface to access subsoil-moisture (Comas et al., 2013). Since the bean grows in sandy soils, water can remain in the root zone for months after rain and a tap root is able to access this water.

Marama bean is also a creeper with scandent stems creeping in several directions covering large areas (Keegan and Van Staden, 1981; **Figure 1A**). This behavior very likely helps to withstand drying winds. Also, typical for a drought avoider, is the relatively early closure of stomata under water stress to save water and maintain the leaf water potential (Mitchell et al., 2005; Karamanos and Travlos, 2012). Stomatal opening represents an important regulatory mechanism during limited water supply and heat stress, influencing simultaneously water loss via transpiration and CO_2_ diffusion into the leaf apoplast. This behavior is very different from a legume like soybean, in which large differences in leaf water potential exists between drought-stressed and unstressed leaves (Villalobos-Rodriquez and Shibles, 1985). Experiments have also been carried out to understand leaf movements in marama bean (Travlos et al., 2008). To avoid direct sunlight, plants can carry out complex daily heliotropic adjustments of leaf angles to reduce transpiration losses by diminishing the light interception (para-heliotropism) in which the *DREB1A* gene might play an important role (van Zanten et al., 2010; Rakocevic et al., 2017). Travlos et al. (2008) reported that the leaves of the bean are open during the day and close during the night, with similar behavior of plants under different growth temperatures. Potassium deficiency can, however, prevent leaf closure during the night but detailed investigations to determine if any heliotropic adjustments are also involved in drought avoidance are so far missing.

A further drought avoidance mechanism of the bean is the formation of a tuberous root (**Figures 1B,C**). The tuber root is both a starch reservoir and a water reservoir. The marama bean root is also high in protein containing about nine percent protein on a dry-weight basis (National Research Council, 2006). A large number of tropical legumes develop below-ground organs for carbohydrate storage (Saxon, 1981). The storage organ may be an enlargement of the tap root, swollen fibrous roots or, as in the case of marama bean, a true tuber. The bean very likely survives a drought condition by accessing the water stored in the tuber. Older tubers weighing more than 200 kg (**Figure 1C**) can contain 90% water by weight. Since the tuber remains viable under drought, it allows rapid vegetative re-growth of stems under more favorable growth conditions as a drought survival strategy (Travlos and Karamanos, 2008). Few older leaves may also maintain leaf function under drought to allow more rapid re-growth when water is again available (Mitchell et al., 2005). However, when the water content of the tuber falls to about 85%, these older leaves are discarded to increase survival for months in a dry environment.

The tuber also has potential as a component of yield. Since marama bean does not flower until the second year after planting from seed, the tuber, if well-developed, can be harvested as a carbohydrate source as well as for starch with interesting properties (Adeboye and Emmambux, 2017; Nyembwe et al., 2018). No detailed information on tuber development is currently available. A different breeding strategy may also be necessary for identifying rapidly developing tubers for harvest as an annual domesticated crop. This also includes a more detailed study on variation in tuber development.

The lack of any root nodules to fix atmospheric nitrogen in marama bean (Dakora et al., 1999) may also have helped evolving in a dry drought-stricken environment. The whole genome assembly from Illumina and PacBio shotgun sequencing has facilitated identification of the marama leghemoglobin genes. These genes are more closely related to the non-symbiotic plant hemoglobin genes rather than the leghemoglobin from symbiotic legumes (Cullis, 2017) supporting an evolutionary hypothesis. Legumes are able to form such root nodules for biological nitrogen fixation facilitated through symbiotic interaction with rhizobia. Although nodulation and atmospheric nitrogen fixation provides a benefit for growth (Morgan et al., 2005), lack of both might be a potential enhancement of drought avoidance. The lack of root nodules avoids dependence on a nitrogen source highly affected by drought conditions. Drought sensitivity of nodules and the particular negative effects on the nodulation process is well known (Serraj et al., 1999; Gil-Quintana et al., 2013). Drought particularly affects supply of photosynthate to nodules, required for symbiotic nitrogen fixation, and also impairs nitrogenase activity with breakdown of the oxygen diffusion barrier and loss of leghemoglobin (King and Purcell, 2006; Arrese-Igor et al., 2011).

## Marama Bean Microbiota

Legumes are generally not highly dependent on atmospheric nitrogen fixation, with both soybean and faba bean the exceptions (Peoples et al., 2009). Legumes can use alternative organic soil nitrogen sources. Soil nitrogen might, for example, derive from compounds such as amino acids (Cao et al., 2016). Such amino acids are found in the rhizosphere as a result of lysis of cells from plants and microbes. Initial experiments to investigate the exact type of soil-derived nitrogen for marama bean have recently been done. Several bacteria have been so far isolated from the rhizosphere of arid-adapted marama bean plants (Kandjimi et al., 2015). All isolates were able to produce ammonia in plate assays. Although ammonia released by bacteria might be a nitrogen source, more information is required about ammonia tolerance of the bean. Plants generally tolerate only low levels of ammonia (Weise et al., 2013). The soil microbiome associated with the bean growing in different regions of Namibia is currently being characterized through population sequencing of the bacterial 16S, V3–V4 region and fungal ITS 1 regions. This will also add to the initial characterization of the endophytes that can be harbored by bean seeds potentially contributing to their nutritional efficiency since they have a striking capacity to harness nitrogen into seed protein (Chimwamurombe et al., 2016). Mycorrhizal fungi are further characterized in the microbiome. These fungi can alleviate drought stress in plants through both tolerance and avoidance mechanisms (Finlay et al., 2008; Rapparini and Peñuelas, 2013). The enhancement of tolerance of plants to water deficit by mycorrhizae may particularly involve the regulation of drought-induced plant genes, such as aquaporins, both by the down-regulation of genes encoding plasma membrane aquaporins (Porcel et al., 2006) or the enhanced expression of specific aquaporins (Li et al., 2012).

## Areas Requiring Further Exploration

Knowledge about avoidance mechanisms in marama bean, but also in other orphan legumes, is still scanty and fragmented and requires more research efforts. Such efforts will not only further drive marama bean breeding but also allow the identification of any correct combination of traits and responses for better survival of a crop in a dry drought-stricken environment. Interesting questions are, for example, why the bean has relatively large leaves (possibly to carry out photosynthesis in a relatively short time) and how the bean maintains, with large leaves, a leaf temperature allowing metabolic processes with Rubisco activase – a key enzyme in keeping the Calvin cycle functional – particularly heat-sensitive (Feller and Vaseva, 2014). This paradox of existence of large-leaf species in deserts, instead of species with small leaves for better reducing any water loss and also intercepting less radiation, is not entirely understood. High rates of leaf transpiration likely provides a significant cooling effect and leaf transpiration is possibly an important trait for surviving in dry and hot environments (Smith, 1978; Chaves et al., 2016; Lin et al., 2017). However, whether this also applies to marama bean has still to be shown.

A further important question is whether marama bean can also use, in addition to drought avoidance, drought tolerance mechanisms with expression of genes providing cellular protection against drought exposure with better water accumulation or can even use drought escape mechanisms (Feller and Vaseva, 2014; Fang and Xiong, 2015; Shavrukov et al., 2017). In this regard, the bean shows no sign of enhanced photosynthetic water-use efficiency at the level of leaf photosynthesis when compared with other well-characterized C3 plants. Rubisco kinetics are further consistent with adaptation to hot, drought-prone environments (Mitchell et al., 2005). Any transcriptomic analysis in the bean is so far also missing to determine, for example, if, and how, genes known to be involved in antioxidant and osmolyte production for drought tolerance are possibly expressed (Hayat et al., 2012; Das and Roychoudhury, 2014). Karamanos and Travlos (2012) found, however, already evidence for a possible progressive osmotic adjustment being more intense in older plants. More negative values of solute potential at zero turgor were thereby considered as an ability of osmotic adjustment through the production, or accumulation, of compatible osmolytes with mobilization of osmotical substances like sugars from the carbohydrates stored in the marama bean root. The Cullis group has therefore recently established a genomic database by high-throughput next-generation sequencing with data from both short read Illumina platform as well as long read PacBio platform. This database, a significant resource in the search for and expression of known protective proteins, now allows the identification and isolation of particular genes known to be involved in drought tolerance. However, an important question remains what benefit a drought tolerance mechanism will have to a plant like marama bean when growing in an dry environment exposed to lengthy drought periods and extensive heat?

A further interesting research topic will be the identification of the actual nitrogen source for the bean. Future bioinformatic studies should therefore also focus on other components of the symbiotic nitrogen fixing pathway to determine whether or not it would be possible for the bean to develop this activity. Symbiosis-related genes might also inform on possible pathways for other bean–microbial interactions in association with information from soil microbiome studies, since there are parallels in the pathways for symbiosis development and mycorrhizal associations. Characterization of the bean’s soil microbiome is consequently an important task, both for nutritional and stress tolerance characters. Interrogation of the genome database for genes necessary for nodulation should further be directed toward identifying rhizobia initiating bean nodulation (De Souza et al., 2015). Finding the nitrogen source(s) for marama bean might also be of relevance for generally growing legumes in dry drought-stricken environments where nodulation for atmospheric nitrogen fixation is severely affected by drought and nitrogen fertilization too costly. Future research also needs to determine if the bean is using a single nitrogen source, such as microbe-derived ammonia, or multiple sources including non-microbial sources. A more detailed investigation of the marama bean microbiome, currently carried out in Namibia and the United States, is therefore an essential task in identification of such nitrogen sources. Marama bean, with its apparently efficient acquisition of nutrition from poor arid soils, might ultimately also provide insights into developing useful microbial fertilizers, especially adapted to arid environments (Chimwamurombe et al., 2016).

Finally, marama bean is an obligate outcrossing species that appears to be functionally hexaploid. Characterization of variation within the germplasm and its relationship to the mechanisms of coping with drought will also identify which genes are important for these processes. Diversity of the bean also indicates that there are possibly differences in growth rate and tuber formation and size. Future identification of interplay of the genome with the microbial associated population will also inform how important relevant genetic variation will be. It has already been shown that the chloroplast genome is variable with individual bean plants heteroplasmic (Kim and Cullis, 2017). Has variability also any relevance to adaptation to a harsh environment is thereby also an important question to answer. Availability of many whole genome sequences for characterized individuals from different environments and the associated microbiome will allow identification of survival/flourishing strategies adopted by the bean and, perhaps, point a way to improving drought tolerance in other crop species.

## Author Contributions

KK and CC developed the first draft to which PC, NB, and JV added to and edited the text. All the authors approved the final submission.

## Conflict of Interest Statement

The authors declare that the research was conducted in the absence of any commercial or financial relationships that could be construed as a potential conflict of interest.

## Footnotes

1 http://www.kirkhousetrust.org/orphanlegumes.html#.V0AvnfmLTIU
